# Different wavelengths of laser: are they significant for treatment of denture stomatitis?: an in-vitro study

**DOI:** 10.1186/s12903-023-03845-8

**Published:** 2024-01-11

**Authors:** Mai Salah El-Din, Ahlam El-Sharkawy, Hams Abdelrahman, Kenda I. Hanno

**Affiliations:** 1 Department of Prosthodontics, Alexandria University Main Hospital, Alexandria, Egypt; 2 Department of Prosthodontics, Faculty of Dentistry, University of Alexandria, Alexandria, Egypt; 3 Department of Dental and Public Health, Faculty of Dentistry, University of Alexandria, Alexandria, Egypt

**Keywords:** Diode laser, Nd-YAG laser, Candida albicans, Growth characteristics, Turbidimetric growth

## Abstract

**Background:**

Denture stomatitis (DS) is an inflammatory disorder that affects the mucosal surface underneath the dentures and frequently causes oral mucosal irritation, discomfort, and altered taste perception, which prevents people from consuming enough nutrients. One of the main causes of DS is an overgrowth of the fungus Candida albicans (C. albicans). A possible alternative treatment for Candida infections is thought to be laser therapy. The aim of this study was to evaluate how different wavelengths of laser would affect growth and pathogenic properties of Candida albicans.

**Methods:**

A concentration of 10^6^ viable cells/ml of Candida albicans were used in the preparation process. Four groups were created from the specimens. Culturing of the control group was completed with no intervention. The other 3 groups received laser radiation for 60 seconds at a power of 1W. The 2^nd^ and 3^rd^ groups were irradiated with diode laser at a wavelength of 940 nm and 980 nm respectively. The 4th group was irradiated with Nd-YAG laser at a wavelength of 1064 nm. Turbidimetric growth was defined as variations in the optical density of fungal growth. These measures were made at three different times: baseline, 48 hours, and 72 hours.

**Results:**

In both groups of diode laser, the growth of Candida albicans showed no remarkable differences at baseline, after 48 and 72 hours using a power of 1 W and duration of 60 seconds. The Nd-YAG group showed significant increase in optical density after 48 hrs then significant decrease after 72 hrs. The optical density values in the control group showed no notable difference between the control and diode study groups at different time periods. However, the Nd:YAG group showed a statistically significant difference compared to the control and the 2 diode laser groups.

**Conclusions:**

Different laser parameters have a different effect on growth and pathogenic properties of Candida albicans. Diode laser therapy with wavelengths 940 and 980 nm used in continuous mode with power of 1 W for duration of 60 seconds can result in proliferation of Candida albicans instead of destroying them. Nd:YAG laser, used in pulsed mode, with power of 1 W for a duration of 60 seconds can be used to destroy Candida albicans and therefore, can be used as an effective treatment for denture stomatitis.

## Background

LASER, stands for “Light Amplification by the Stimulated Emission of Radiation,” was first used by Gordon Gould in 1959, a graduate student at the educational institution of Columbia [1]. The first operational laser was created by Theodore Maiman at the research facilities of Hughes in Malibu, California, using a combination of helium and neon [2]. A laser named neodymium-doped yttrium aluminum garnet (Nd:YAG) was created in 1961 from yttrium-aluminum-garnet crystals treated with 1-3% neodymium [1].

In the following year, argon laser was created followed by the ruby laser in 1963 which was the first laser to have medical applications such as coagulation of lesions in the retina [1]. In 1964, Patel created the CO2 laser in 1964. In the field of dentistry today, diode lasers are widely employed [1].

There are several ways to categorize lasers used in dentistry practice: According to the type of laser being used, such as a gas laser or a solid laser; the type of tissue to which the laser can be applied, such as a hard tissue laser or a soft tissue laser; the range of wavelengths; and of course, the danger connected with using a laser [3].

Diode laser has gained popularity in dentistry, due to its compact size and ease of use for surgical procedures of minor tissues [4]. The ablation/vaporization method removes the oral mucosal lesions based on the diode laser’s photothermal effect [5].

The diode laser’s active medium, a semiconductor composed of gallium, aluminium, arsenide, and sporadically indium, generates wavelengths of laser that fall between 810 nm and 980 nm. Hemoglobin and tissue melanin absorb most of all diode wavelengths [6]. In contrast, the components of enamel, which include water and hydroxyapatite, have a difficult time absorbing them. Aesthetic recontouring of the gingiva, exposing teeth impacted in soft tissue, removal of frenum, removing inflammatory and enlarged tissue, soft tissue crown lengthening, and treatment of apthous and herpetic lesions are some of the specific applications of diode laser [6].

The near-infrared region of the electromagnetic spectrum, is where the 1064 nm wavelength of the Nd:YAG laser is located [7]. There are different applications of Nd:YAG laser in the literature [7, 8]. Previous studies investigated the antibacterial properties of the Nd:YAG laser and how well it can reduce endodontic biofilms and periodontal inflammation [9, 10].

The oral cavity of humans can become infected by several distinct species of Candida. Oral candidiasis is most frequently caused by Candida albicans (C. albicans) [11]. Old age, prolonged use of corticosteroids, diabetes, transplanting of organs, intake of broad-spectrum antibiotics, and wearing dentures are a few circumstances that raise a person’s risk of oral candidiasis [12, 13]. As the population ages and the number of persons with weakened immune systems rises, candidiasis and the related illness have increased [14–18].

Denture stomatitis, a persistent inflammatory illness that is more common in the mucosa of the palate of persons using poorly fitting dentures, can be brought on by Candida [19]. Between 33% and 67% of patients who wear complete dentures experience this issue [20]. As a practical first step in the development of denture stomatitis, C. albicans may adhere to both mucosal and denture surfaces.

Denture stomatitis is thought to affect more women than men [21]. The multifaceted nature of the condition makes treating denture stomatitis somewhat challenging. Improved dental hygiene, the use of disinfectant mouth rinse, nocturnal removal of the dentures and soaking them in a sanitizing solution and changing poorly fitting dentures are all examples of conventional therapies [22].

Candida colonization has lately been thought to be prevented by diode laser, whose effects are based on the decreased porosity of denture tissue surface [23–30]. Other studies [31–33] focused more on the treatment of denture stomatitis than on its prevention. According to Basso FG et al., low level laser treatment (LLLT) has an inhibitory effect on microorganisms, and the strength of this effect can vary depending on how various microbial species interact with one another [34]. Recent studies concluded that Nd:YAG laser has reduced the number of cells and cell metabolism of C.albicans [35, 36].

Previous research has shown that different lasers are efficient against germs and fungi [37, 38], however, nothing is known about how the Nd:YAG laser affects fungi’s ability to change their physiological and biochemical features. There is no sufficient evidence on the most effective laser protocol to eradicate C. albicans.

The present study aimed at comparing the effects of diode laser (940 and 980 nm) and Nd:YAG (1064 nm) laser on the growth characteristics and pathogenic properties of C.albicans. The null hypothesis was that two wavelengths of diode (940 and 980 nm) and Nd:YAG laser (1064 nm) would not have a different effect on the colonization of C. albicans.

## Methods

The Committee of Research Ethics in Alexandria University, Faculty of Dentistry (IORG 0008839) has approved the research prior to any research-related activities.

The sample size was calculated using 5% alpha error and 80% study power [39]. Nine specimens were required with an effect size of 1.44. This was increased to 10 specimens in each group to make up for procedures errors. Total sample was equal to 40 specimens. Based on Rosner’s technique [40], the sample size was obtained by G*Power software version 3.1.9.7 [41].

### Specimens’ preparation

In this study a total of 40 specimens were allocated into a control group, a diode 940 nm group, and a diode 980 nm group and Nd:YAG 1064 nm group.

A strain of C. albicans (ATCC 90028) was purchased from the Microbiology Department, Faculty of Medicine, Alexandria University, Egypt. The specimens were initially diluted with sterile saline (0.85% NaCl) to obtain concentration of 1.5 × 10^6^ live cells/ml in order to prepare the strain’s particular concentrations. The specimens were grown on Sabouraud dextrose agar with concentration set to standard solutions of McFarland turbidity of 0.5 [42].

The chosen isolate of fungal cells was employed throughout the study to offer standardization after a pure culture had been established. Then, colonies of C. albicans were allocated to control and treatment plates, leaving a single colony with a surface area of less than 1 cm^2^ on each plate [43]. All of the produced specimens were then set aside and incubated for 24 hours at 37 °C [42].

### Control group

**Fig. 1:**
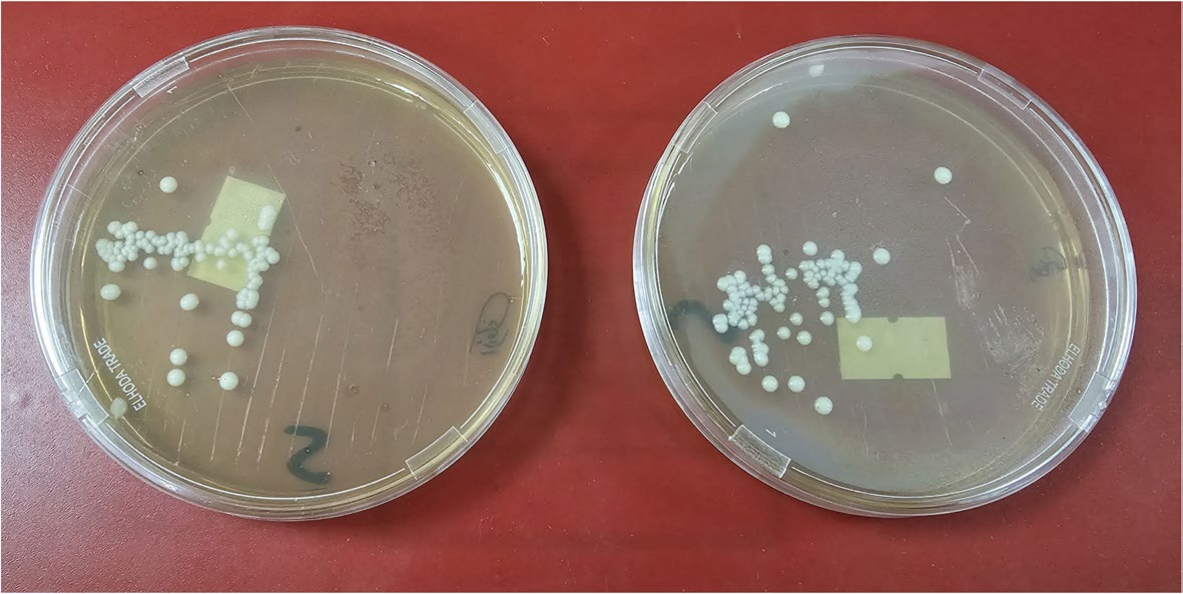
Candida colonies on agar plates

The control group included 10 specimens. Culturing of the specimens was done to make sure that the C.albicans will grow in vitro (Fig. 1). No laser irradiation was done in the control group.

### Diode 940 nm group

**Fig. 2:**
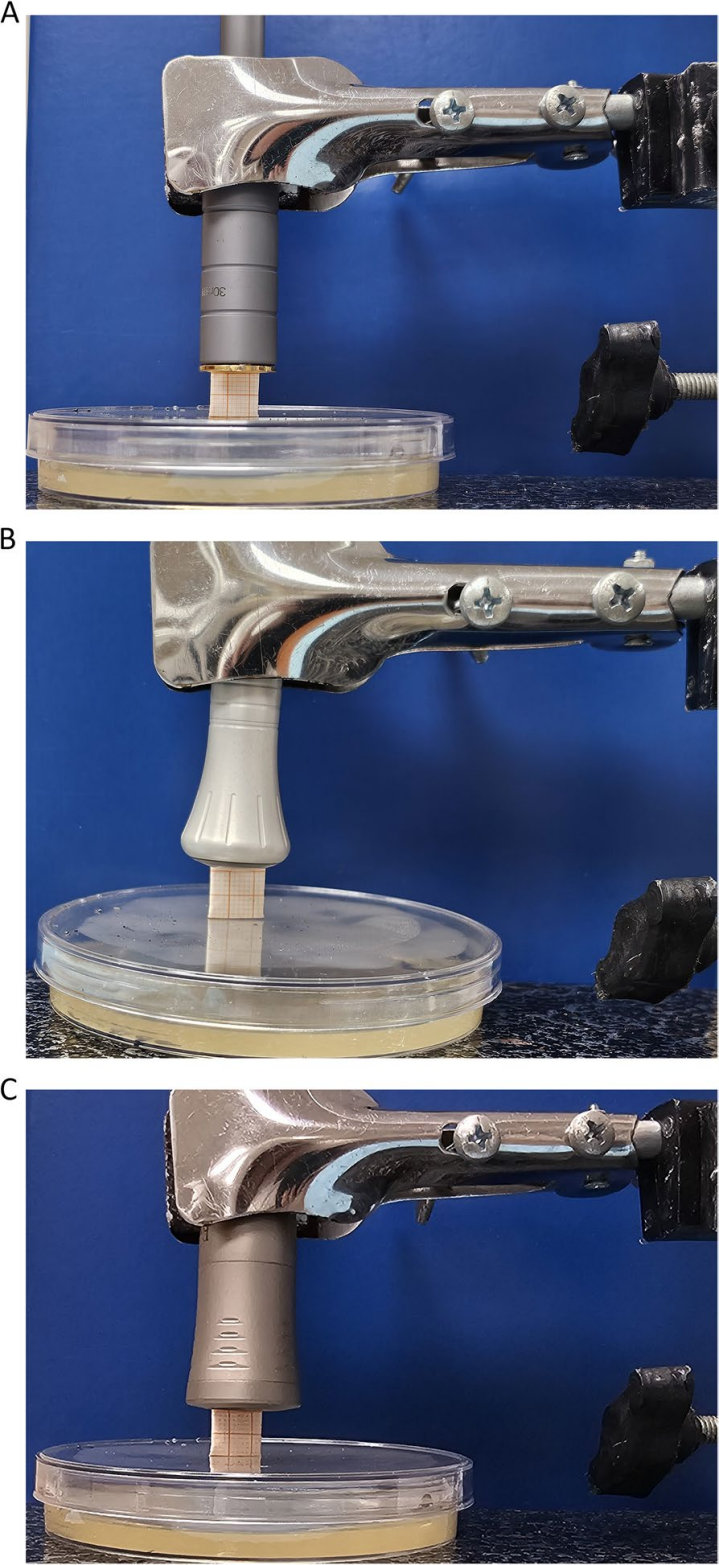
Laser irradiation of Candida albicans. **A** Irradiation with diode 940 nm. **B** Irradiation with diode 980 nm. **C** Irradiation with Nd:YAG 1064 nm

The 940 nm diode laser group consisted of 10 specimens that were exposed to diode laser therapy at wavelength 940nm (Epic 10; Biolase Inc.) with a power of 1W, energy density of 60 J/cm^2^ and duration of60 seconds in continuous mode, using the deep tissue handpiece (Fig. 2A) [44].

### Diode 980 nm group

The 980 nm diode laser group consisted of 10 specimens that were exposed to diode laser therapy at wavelength 980 nm (SMART M, Lasotronix, Piaseczno.) with a power of 1W, energy density of 60 J/cm^2^, and duration of 60 seconds [42] in continuous mode with a flat-top handpiece with a tip diameter of 18 mm (Fig. 2B).

### Nd:YAG 1064 nm group

The 1064 nm Nd: YAG group consisted of 10 specimens that were exposed to Nd:YAG at wavelength 1064 nm (LightWalker Line, Fotona) with a power of 1W, energy density of 60 J/cm^2^, duration of 60 seconds, frequency of 10 Hz, with a flat-top handpiece (Genova, LightWalker, Fotona) with a spot diameter of 11 mm in non-contact pulsed mode, [35] (Fig. 2C).

The laser didn't need to be calibrated because it was preconfigured. According to the guidelines specified for the study, the laser head was supported by a device that is specifically made to deliver homogeneous laser irradiation from outside the culture plates’ walls on which seeding of the microorganisms is accomplished. The beam of diode laser was transmitted 1 cm over the surface of the colonies through the glassy cover of the plate to prevent contamination [43]. Prior to the necessary treatments, all specimens were coded [42]. The operators put on protective eyewear before beginning the procedure [45].

### Quantifying turbidimetric growth

The turbidimetric growth rates of both the control and study groups were calculated [45–48]. To make the inocula, a culture was diluted with 0.9% NaCl to 1.5x10^6^ CFU/mL and left overnight. To achieve a final inoculum between 0.5 and 2.5x10^3^ CFU/mL, the yeast suspensions were further diluted in brain heart broth (Brain Heart Infusion, OXOIDTM, CM1135B) [43].

**Fig. 3:**
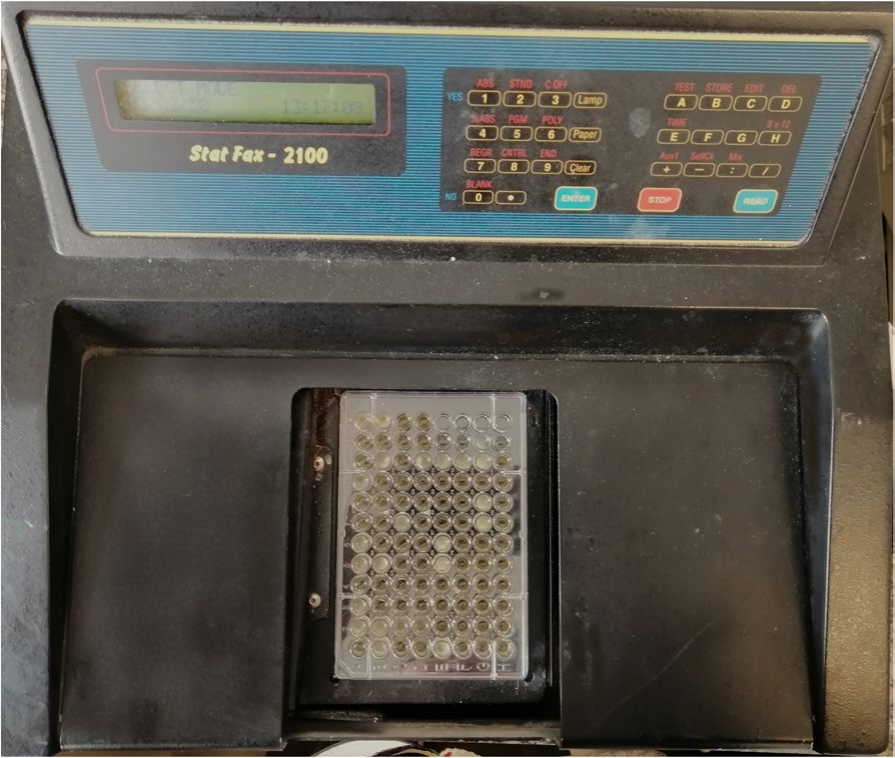
Measurement of turbidimetric growth of Candida albicans

A sterile flat-bottomed microtiter plate consisting of 96 wells, with brain broth (100 μL/well) was filled with 100 μL of fungal inocula (Fig. 3). For each isolate, inoculations were carried out twice, with two wells containing nothing but media (background control) [43].

Following inoculation, the plate was incubated for 72 hours at room temperature inside a microplate reader (Awareness Technologies Inc. Stat Fax 2100) at 37°C to measure the suspension's absorbance at 630 nm. Without shaking, the optical density at 630 nm (OD630) was captured for each well [48, 49]. At baseline, 48 hours, and 72 hours following the application, evaluations were conducted. The variation of OD630 over time was investigated [43]. Evaluation was done blindly by a single trained operator to avoid evaluation bias.

Normality was tested using the Shapiro Wilk test and normality plots. The Kruskal Wallis test was used to compare between groups followed by Dunn’s post hoc test with Bonferroni correction and Friedman test was performed to compare between time intervals within each group. The significance level was set at *P* value=0.05 and all tests were two tailed. Analysis of data was completed using IBM SPSS version 23, Armonk, NY, USA.

## Results

In this study, a total of 40 specimens were allocated into a control group, diode 940 nm group, diode 980 nm group, and Nd:YAG (1064 nm) group.

**Fig. 4:**
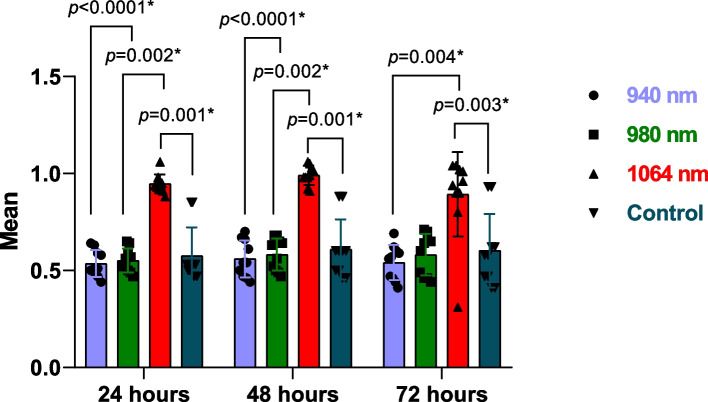
Box plot diagram showing the optical density of Candida albicans and pairwise comparisons at different time points

Table 1 and Fig. 4 show comparison of candida colonies among the study groups at different time points. Analysis of optical density of candida albicans’ colonies showed a statistically significant difference between the study groups. At the different three time points, the highest median optical density was among 1064 nm Nd:YAG group while 940 nm diode group showed the lowest optical density.

**Table 1:** Comparison of candida colonies among the study groups at different time points

		**940 nm**	**980 nm**	**1064 nm**	**Control**	**H Test** **(*p* value)**
Baseline	Median (Min – Max)	0.509 (0.437 – 0.640)^a^	0.540 (0.472 – 0.650)^a^	0.940 (0.879 – 1.056)^b^	0.529 (0.473 – 0.850)^a^	22.168 (<0.0001*)
48 hours	Median (Min – Max)	0.537 (0.437 – 0.695)^a^	0.592 (0.472 – 0.677)^a^	0.986 (0.912 – 1.058)^b^	0.595 (0.460 – 0.884)^a^	22.207 (<0.0001*)
72 hours	Median (Min – Max)	0.563 (0.412 – 0.688)^a^	0.619 (0.443 – 0.709)^ab^	0.950 (0.309 – 1.043)^b^	0.584 (0.412 – 0.928)^a^	13.484 (0.004*)
**Test** **(*p* value)**		1.947 (0.378)	2.526 (0.283)	11.128 (0.004*)	3.200 (0.202)	
**Pairwise comparisons**		-	-	*p*_1_=0.011, *p*_2_=1.00, *p*_3_=0.016*	-	

^*^Statistically significant difference at *p* value≤0.05, superscript different letters denote statistically significant difference between the groups, *p*_1_: comparison between baseline and 48 hours, *p*_2_: comparison between baseline and 72 hours, *p*_3_: comparison between 48 hours and 72 hours

According to pairwise comparisons, the 940 nm diode group and the 1064 nm Nd:YAG group differed significantly at baseline, after 48, and after 72 hours. The 980 nm group showed a statistically significant difference compared to the 1064 nm Nd:YAG group at baseline, and 48 hours. The 1064 nm Nd:YAG group showed a significant difference in comparison to the control group in the three time periods.

In the 940 nm, and 980 nm diode laser groups, the median optical density of Candida albicans’ colonies increased after 48 hours and 72 hours, however, there was no significant difference between the three time periods (*P*=0.378, and 0.283, respectively). Whereas, in the 1064 nm Nd:YAG laser group, the median optical density of the candida colonies increased significantly at 48 hours (*P*=0.011) then it decreased significantly again at 72 hours (*P*=0.016). A similar pattern was observed for the control group with no difference between the three time points (*P*=0.202).

## Discussion

Previous literature has not found an explanation of how the diode laser of a wavelength of 980 nm has affected the biofilm of C. albicans. Several researches have examined how bacteria are affected by lasers, the results suggest to be applied to C. albicans [27].

In this study, we have evaluated the turbidimetric growth of C. albicans which is an indication of how the growth was affected after exposure to 940 nm, 980 nm diode laser, and 1064 nm Nd-YAG laser.

Seyedmousavi et al studied the effect of low-level laser therapy of wavelength 685 nm with different applied energies of 3, 5, 10, and 20 J and a wavelength of 830 nm with energies of 3, 5, 10, 30, and 50 J. In vitro evaluation showed significant effects on the turbidimetric growth kinetics. They concluded that certain wavelengths of laser can reduce the pathogenic properties of C. albicans without the need for photosensitizing dye, therefore, laser can be used to treat C. albicans infections [43].

In the present study, there were no significant effects in the changes in the optical density of the C. albicans in both 940 nm and 980 nm diode laser over the 3 time periods compared to the control group. However, the values increased with time indicating that the diode laser of both wavelengths enhanced the growth of C. albicans, which follows the results of Najafi et al [42], who compared the effect of using nystatin with the use of diode laser therapy of a wavelength of 940 nm and a power of 1W for a duration of 30 s or 60s in a continuous mode on the growth of Candida albicans, and found that the 940nm diode laser caused an increase in C. albicans colonies [42]. This could be because laser therapy is known to enhance the electron transfer process in the mitochondria which results in production of Adenosine triphosphate (ATP) [25].

The results of our study are also similar to Carneiro et al, who investigated how laser irradiation affected C.albicans [26]. They used laser with wavelength of 830 nm and a power of 40mW and a wavelength of 685 nm and a power of 30mW with energy values of 6,8,10, and 12 J/cm2 and found no difference in the number of colonies of C. albicans [26].

However, this result is opposite to the results by Sennhenn-Kirchner et al who investigated the efficacy of erbium:yttrium-aluminium-garnet (Er:YAG) light (2940 nm) and diode laser light (810 nm) by comparing with the control groups which were irradiated, and concluded that the Candida albicans’ cells were significantly reduced by both types of lasers. However, the Er:YAG revealed a greater effect compared to the diode laser [24].

The results may be related to different parameters such as energy levels, power, duration of exposure, laser wavelength and method of laser irradiation, whether it is contact or non-contact, pulsed or continuous mode. In this study, continuous mode was used. It was stated that in continuous mode, the thermal energy is distributed which prevents excess heat from accumulating in the tissues, while in pulsed mode, higher heat levels accumulate inside the tissues thus causing more tissue damage [23].

In this study, the 1064 nm Nd:YAG group showed a statistically significant difference in comparison to the control group and to both wavelengths of diode laser in the 3 different time periods. There was a significant increase in the optical density of the candida colonies in the Nd-YAG laser group after 48 hours, however, the optical density then decreased after 72 hours, and the difference was statistically significant. This is consistent with the finding of Kinga Grzech-Leśniak et al [35] who claimed that both C. albicans and S. mutans' cfu/ml values decreased as a result of being exposed to Nd:YAG laser radiation using a flat-top handpiece. These results show that the infrared wavelength of 1064 nm is effective and secure in lowering the number of irradiated microorganisms.

They also reached a conclusion that more cell debris could be visible following the disintegration of cells exposed to greater power and longer Nd-YAG laser exposure times, suggesting that lasers may be more successful at destroying cells than at inhibiting their activity. The cells that were still active following the laser treatment tended to alter form, elongate, and prepare to assume different morphological structure [35].

Clinicians should consider the different parameters of laser and their effects on C. albicans for the management of denture stomatitis. The limitations of our study included that only 3 wavelengths of laser were investigated with set parameters. Further research is necessary to evaluate the effect of different energy densities, power, and duration of laser irradiation using laser therapy on growth and pathogenicity of C. albicans.

## Conclusions

The effects of diode laser therapy on C. albicans and other infectious agents depend on several factors which include the type and wavelength of laser therapy, the laser parameters used including the power, energy, duration of irradiation, pulsed or continuous mode, contact or non-contact mode and distance from the exposed area. Diode laser of wavelengths 940 nm and 980 nm, used in a continuous mode with power of 1 W and a duration of 60 seconds had no significant effect on the C. albicans, while Nd:YAG laser of wavelength 1064 nm, used in pulsed mode with a power of 1 W for 60 seconds, was effective in reducing C. albicans and therefore can be used for management of denture stomatitis.

## Data Availability

The datasets used and/or analysed during the current study are available from the corresponding author on reasonable request.

## Abbreviations

DS: Denture Stomatitis
C.albicans: Candida albicans
LLLT: Low Level Laser Therapy
CFUs: Colony-Forming Units
OD630: Optical Density at 630 nm
Er:YAG: Erbium:Yttrium Aluminium-Garnet
Nd:YAG: Neodymium-doped: Yttrium Aluminum Garnet
